# Differential temporal profile of lowered blood glucose levels (3.5 to 6.5 mmol/l versus 5 to 8 mmol/l) in patients with severe traumatic brain injury

**DOI:** 10.1186/cc6974

**Published:** 2008-08-04

**Authors:** Regula Meier, Markus Béchir, Silke Ludwig, Jutta Sommerfeld, Marius Keel, Peter Steiger, Reto Stocker, John F Stover

**Affiliations:** 1 Surgical Intensive Care Medicine, University Hospital Zuerich, Raemistrasse 100, CH 8091 Zuerich, Switzerland; 2 Department of Surgery, Division of Trauma Surgery, University Hospital Zuerich, Raemistrasse 100, CH 8091 Zuerich, Switzerland

## Abstract

**Introduction:**

Hyperglycaemia is detrimental, but maintaining low blood glucose levels within tight limits is controversial in patients with severe traumatic brain injury, because decreased blood glucose levels can induce and aggravate underlying brain injury.

**Methods:**

In 228 propensity matched patients (age, sex and injury severity) treated in our intensive care unit (ICU) from 2000 to 2004, we retrospectively evaluated the influence of different predefined blood glucose targets (3.5 to 6.5 versus 5 to 8 mmol/l) on frequency of hypoglycaemic and hyperglycaemic episodes, insulin and norepinephrine requirement, changes in intracranial pressure and cerebral perfusion pressure, mortality and length of stay on the ICU.

**Results:**

Mortality and length of ICU stay were similar in both blood glucose target groups. Blood glucose values below and above the predefined levels were significantly increased in the 3. 5 to 6.5 mmol/l group, predominantly during the first week. Insulin and norepinephrine requirements were markedly increased in this group. During the second week, the incidences of intracranial pressure exceeding 20 mmHg and infectious complications were significantly decreased in the 3.5 to 6.5 mmol/l group.

**Conclusion:**

Maintaining blood glucose within 5 to 8 mmol/l appears to yield greater benefit during the first week. During the second week, 3.5 to 6.5 mmol/l is associated with beneficial effects in terms of reduced intracranial hypertension and decreased rate of pneumonia, bacteraemia and urinary tract infections. It remains to be determined whether patients might profit from temporally adapted blood glucose limits, inducing lower values during the second week, and whether concomitant glucose infusion to prevent hypoglycaemia is safe in patients with post-traumatic oedema.

## Introduction

After severe traumatic brain injury (TBI), secondary brain damage related to activated local cascades as well as systemic influences can compromise regenerative and reparative processes, thereby increasing morbidity and mortality. Within this context, elevated blood glucose concentrations at admission and during intensive care exceeding 9.4 mmol/l (170 mg/dl) are associated with increased mortality [1,2] and morbidity [3-5] compared with normoglycaemic patients. Consequently, it appears logical to correct and maintain blood glucose levels at lower yet controllable values in order to prevent and counteract hyperglycaemia-induced mitochondrial damage, sustained cytotoxic oxidative stress, impaired neutrophil function and reduced phagocytosis, as well as impaired intracellular bactericidal and opsonic activity [6].

As recently shown by van den Berghe and colleagues [7], maintaining blood glucose levels at low levels ranging from 4.4 to 6.1 mmol/l (80 to 110 mg/dl), as compared with concentrations exceeding 12 mmol/l (220 mg/dl), appears to be beneficial for surgical and medical patients requiring intensive care treatment longer than 3 days. Overall, this approach significantly reduced morbidity and mortality, and prevented critical illness polyneuropathy, bacteraemia, anaemia, acute renal failure and hyperbilirubinaemia. These benefits ultimately culminated reduced length of hospitalization, duration of ventilation and substantially lowered costs [7].

Patients with various types of traumatic and nontraumatic brain lesions also appear to profit from this approach [8]. However, this reduced infection rate and mortality could not be reproduced by Bilotta and colleagues [9] in their prospective randomized trial conducted in brain-injured patients employing a similar study design to that used by van den Berghe and colleagues [7].

Following the results published by van den Berghe and colleagues [7], targeted blood glucose levels were lowered from 5 to 8 mmol/l (90 to 144 mg/dl) to 3.5 to 6.5 mmol/l (63 to 117 mg/dl) at our institution, with the aim being to reduce cellular insults related to high blood glucose levels and concomitantly to promote insulin-mediated nonglycaemic protective effects related to the anti-apoptotic and anti-inflammatory effects of normoglycaemia.

Recently, implementation of these tightly controlled blood glucose levels was criticized in brain-injured patients because of the resulting increased risk for hypoglycaemic episodes, which promote an increase in extracellular glutamate and signs of metabolic derangement, reflected by an increased lactate/pyruvate ratio [10]. Absolute as well as relative decreases in blood glucose concentrations below 5 mmol/l were consistently associated with spontaneous cortical depolarizations under both experimental and clinical conditions [11-14]. These alterations with and without excessive correction of hypoglycaemic values are in turn feared to induce secondary brain injury, thereby possibly offsetting anticipated neuroprotection in these patients.

The main hypothesis of the present study was that maintaining arterial blood glucose between 3.5 to 6.5 mmol/l, as compared with 5 to 8 mmol/l, significantly decreases mortality and reduces rates of infectious complications. Based on this hypothesis, primary end-points were intensive care unit (ICU) mortality, and rates of pneumonia, bacteraemia and urinary tract infections. In addition, we investigated the impact of maintaining blood glucose levels within low and tight limits on fluctuations in blood glucose values, insulin and norepinephrine requirements, alterations in intracranial pressure (ICP), length of stay on the ICU, and signs of inflammation in patients with severe TBI. For this, we retrospectively compared 114 propensity-matched patients in whom blood glucose levels were maintained between 3.5 to 6.5 mmol/l with 114 patients with a blood glucose target between 5 to 8 mmol/l. Patients were matched with respect to age, sex, and type, number and severity of injuries.

## Materials and methods

**Figure 1:**
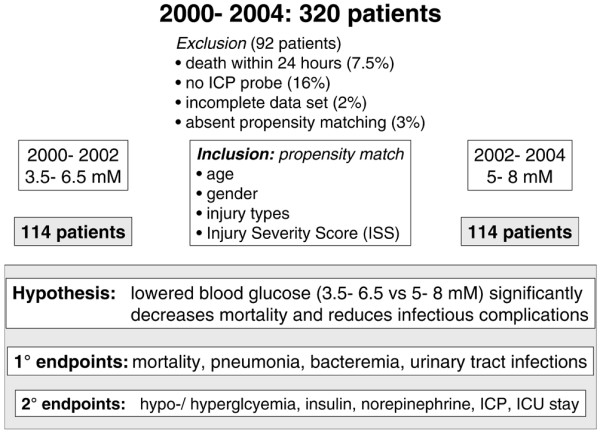
Study description. Presented is a flow chart showing inclusion of 228 patients and exclusion of 92 patients suffering from severe traumatic brain injury subjected to two different blood glucose targets, namely 3.5 to 6.5 mmol/l versus 5 to 8 mmol/l, over a period of 4 years. The main hypothesis as well as primary and secondary end-points are shown.

Following approval by the local ethics committee, which waived the need for written informed consent for this retrospective study, patient records from a total of 320 patients treated on our ICU from 2000 to 2004 were reviewed. In the years 2000 to 2002, blood glucose levels were maintained between 4 and 8 mmol/l. Thereafter, blood glucose limits were reduced to 3.5 to 6.5 mmol/l during the years 2002 to 2004. Following exclusion of 92 patients (29%), 228 propensity-matched critically ill patients suffering from severe TBI were eligible for subsequent analysis aimed at comparing the influence of blood glucose levels maintained between 3.5 to 6.5 mmol/l versus 5 to 8 mmol/l (Figure 1).

### Propensity-matched patients

To increase comparability between patients who were treated sequentially (2000 to 2002 and 2002 to 2004) with different blood glucose limits, patients were matched according to age, sex, injury types and severity of underlying injuries based on the Injury Severity Score, determined after admission to the emergency room of the University Hospital Zuerich. This allowed us to minimize the impact of uncontrolled influences that can occur over a 4-year period.

### Inclusion criteria

Patients had to be treated on our ICU for longer than 24 hours. All patients were required to have had an ICP probe placed within the first 8 hours after injury.

### Exclusion criteria

Patients who died within the first 24 hours after injury and those in whom an ICP probe was not inserted (low or high severity of injury) were not included. Patients with incomplete data were excluded as well.

### Standardized critical care

All patients were treated using a standardized protocol. Analgesia and sedation were maintained with fentanyl (Sintenyl^®^) and midazolam (Dormicum^®^). If required, muscle relaxation was induced with pancuronium. Haemodynamic stability was maintained by fluid and vasopressor administration and adapted to maintain cerebral perfusion pressure (CPP) between 70 and 90 mmHg. Increased analgesia, sedation, CPP, controlled hyperventilation and cerebral spinal fluid release in patients with external ventricular drainage were employed to maintain ICP levels below 20 mmHg. Lung protective ventilation was maintained by keeping peak inspiratory pressure below 35 mbar. Enteral nutrition was begun within the first 12 hours and controlled by means of indirect calorimety at least twice weekly. Continuously infused insulin was tapered according to the measured blood glucose levels. Contrary to the protocol used by van den Berghe and colleagues [7], we did not routinely infuse glucose in our patients because of concern that increased post-traumatic brain oedema formation might result. Glucose was only infused in case of hypoglycaemia under 1.5 mmol/l. Blood glucose levels were determined using the blood gas analyzer ABL 825 Flex (Radiometer, Copenhagen, Denmark) at least every 4 hours or at shorter intervals, depending on the clinical situation and the determined blood glucose level, in order to avoid hypoglycaemic and hyperglycemic episodes. Hypoglycaemia was defined at blood glucose levels under 2.5 mmol/l, whereas hyperglycaemia was defined at blood glucose concentrations above 10 mmol/l.

### Investigated parameters

The data bank (Microsoft Excel^® ^and Microsoft Access^®^; Microsoft Inc., Redmond WA, USA) consisted of values that were determined at 4-hour intervals: blood glucose, infused insulin and norepinephrine dose, as well as ICP and CPP levels. In addition, mortality, length of ICU stay, positive blood cultures and positive tracheobronchial secretions, as well as changes in maximal leukocytes, C-reactive protein (CRP) and interleukin-6 (IL-6), were recorded. This resulted in a total of 58,794 values in all patients and an average of 258 values per patient.

Values assessed at 4-hour intervals or once daily were used to determine changes in the individual parameters over time and to calculate absolute and relative frequencies within predefined clusters.

The database was constructed by entering data in predefined columns within a Microsoft Excel^® ^sheet for every individual patient. Then, all individual sheets were transferred to one Microsoft Excel^® ^sheet, which contained data for all patients. This Microsoft Excel^® ^sheet was then imported into a Microsoft Access^® ^database. Data were entered by RM, SL and JS, and checked for plausibility and correctness by JFS and SL; after an automated search for incorrect outliers within each column, these values were then corrected by referring to the original patient records.

Relative frequency was determined by first assessing the absolute number of values found within predefined clusters, followed by expressing the number of values or incidences per predefined cluster as a percentage of the absolute number of all values of a certain parameter, for instance arterial blood glucose.

Blood glucose variability was assessed by calculating the arithmetic difference compared with the previous arterial blood glucose value.

### Statistical analysis

Changes over time and between groups were evaluated for statistically significant difference using the Mann-Whitney rank sum test and analysis of variance on ranks. Survival probability was determined by log-rank analysis (Kaplan-Meier survival analysis with surviving patients subjected to censoring). *P *< 0.05 was considered to represent statistical significance. Statistical analysis was performed using SigmaStat^®^3.5; figures were created using SigmaPlot^®^10.0 (SYSTAT Software Inc., Swtizerland)

## Results

### Demographic data and mortality

**Figure 2:**
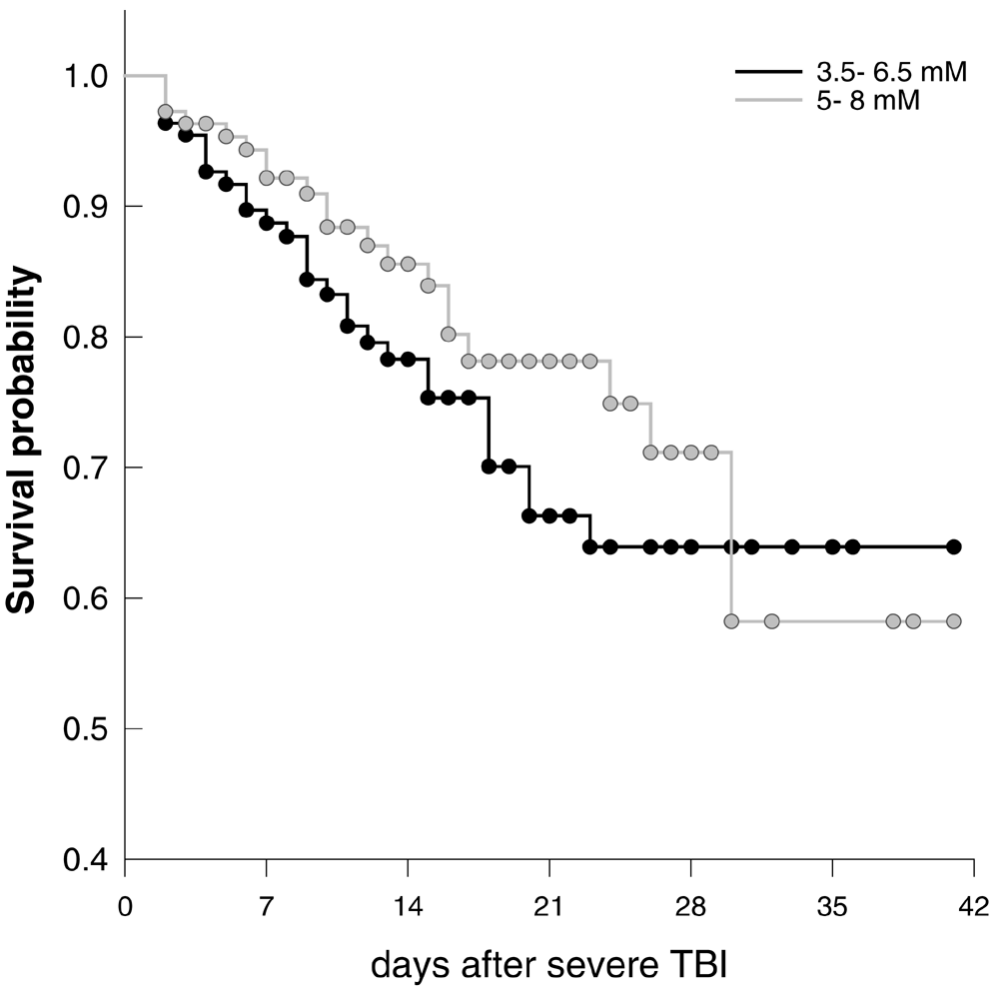
Survival during the first 2 weeks. The Kaplan-Meier survival curve illustrates a trend toward increased mortality during the first 2 weeks in patients subjected to blood glucose target of 3.5 to 6.5 mmol/l compared with 5 to 8 mmol/l.

Propensity-matched patients (Table 1) within the 3.5 to 6.5 mmol/l blood glucose group exhibited a nonsignificant trend toward an increased mortality rate during the first 2 weeks compared with the 5 to 8 mmol/l group (Table 1 and Figure 2). Overall mortality rates were 25% versus 19% (3.5 to 6.5 mmol/l versus 5 to 8 mmol/l). There was no significant difference between groups.

**Table 1:** Demographic data

Parameters	Blood glucose 3.5 to 6.5 mmol/l	Blood glucose 5 to 8 mmol/l
Number of patients	114	114
Men (*n *[%])	87 (76%)	87 (76%)
Women (*n *[%])	27 (24%)	27 (24%)
Isolated TBI (*n*)	40	40
Multiple injuries (*n*)	74	74
Age (years; mean [range])	41 (18–81)	38 (18–81)
AIS head (mean [range])	25 (9–36)	25 (9–36)
AIS without head (mean [range])	16 (1–55)	16 (1–55)
ISS (mean [range])	34 (16–54)	34 (12–67)
Injured organs (*n *[range])	2 (1–5)	2 (1–5)
Initial GCS (mean [range])	11 (3–15)	10 (3–15)
CT lesions (n [%])		

EDH	4 (3.5%)	3 (2.6%)
SDH	8 (7%)	12 (10.5%)
Contusions	15 (13.2%)	15 (13.2%0
Generalized oedema	7 (6.1%)	9 (7.9%
tSAH	3 (2.6%)	6 (5.3%)
mixed lesions	77 (67.5%)	69 (60.5%)

Surgery (%)		

ICP probe	100%	100%
Fractures	28%	31%
Craniectomy	4%	4%

ICP > 20 mmHg (*n *[%])	27 (24%)	33 (29%)
Nonsurvivors (*n *[%])	12 (41%)	11 (50%)
Survivors (*n *[%])	22 (26%)	17 (18%)

Arterial hypotension (SBP < 90 mmHg; *n*)	1	1

Mortality (*n*/*n *[%])	29/114 (25%)	22/114 (19%)
Week 1	12/114 (11%)	8/114 (7%)
Week 2	9/89 (10%)	5/86 (6%)
Week 3	8/57 (14%)	9/57 (16%)

ICU length (days; median [range])		

Survivors	17 (2–48)	15 (2–52)
Deceased	9 (2–23)	11 (2–43)

Demographic data in 228 propensity-matched patients with severe traumatic brain injury (TBI) subjected to two different blood glucose targets: 3.5 to 6.5 mmol/l versus 5 to 8 mmol/l. AIS, abbreviated injury score; CT, computed tomography; EDH, epidural haematoma; GCS, Glasgow Coma Scale; ICP, intracranial pressure; ICU, intensive care unit; ISS, injury severity score; SDH, subdural haematoma; tSAH, traumatic subarachnoid haemorrhage; BP = blood pressure.

### Influence of additional injuries

Presence, type and degree of intracranial and extracranial injuries had no statistically significant influence (data not shown). Thus, TBI patients with and without additional injuries were combined for subsequent analysis.

### Changes in blood glucose levels

**Figure 3:**
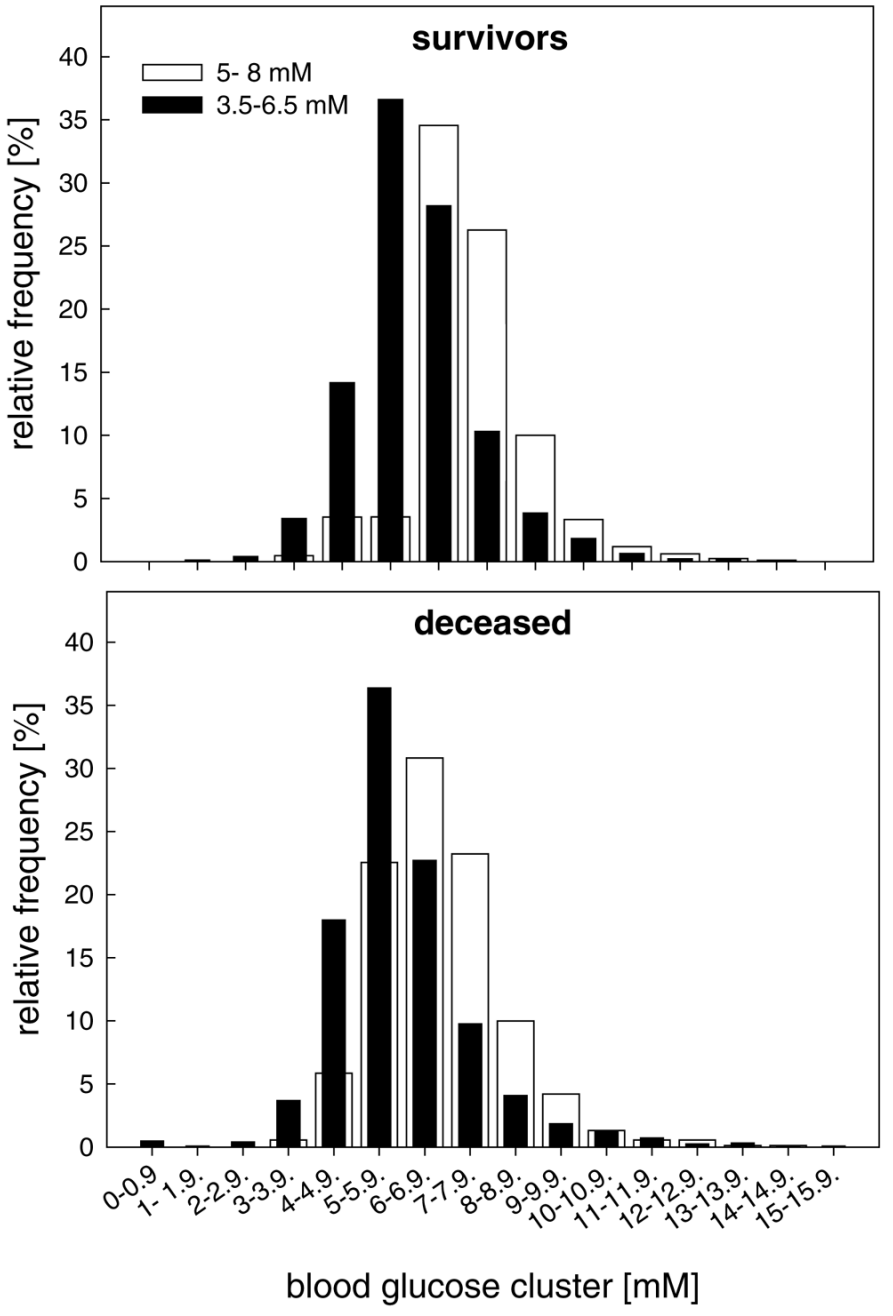
Arterial blood glucose levels. Presented are histograms showing distribution of arterial blood glucose levels within predefined clusters in surviving patients (upper panel) and patients who died (lower panel) treated within the 3.5 to 6.5 mmol/l (black columns) and 5 to 8 mmol/l (white columns) blood glucose targets.

**Figure 4:**
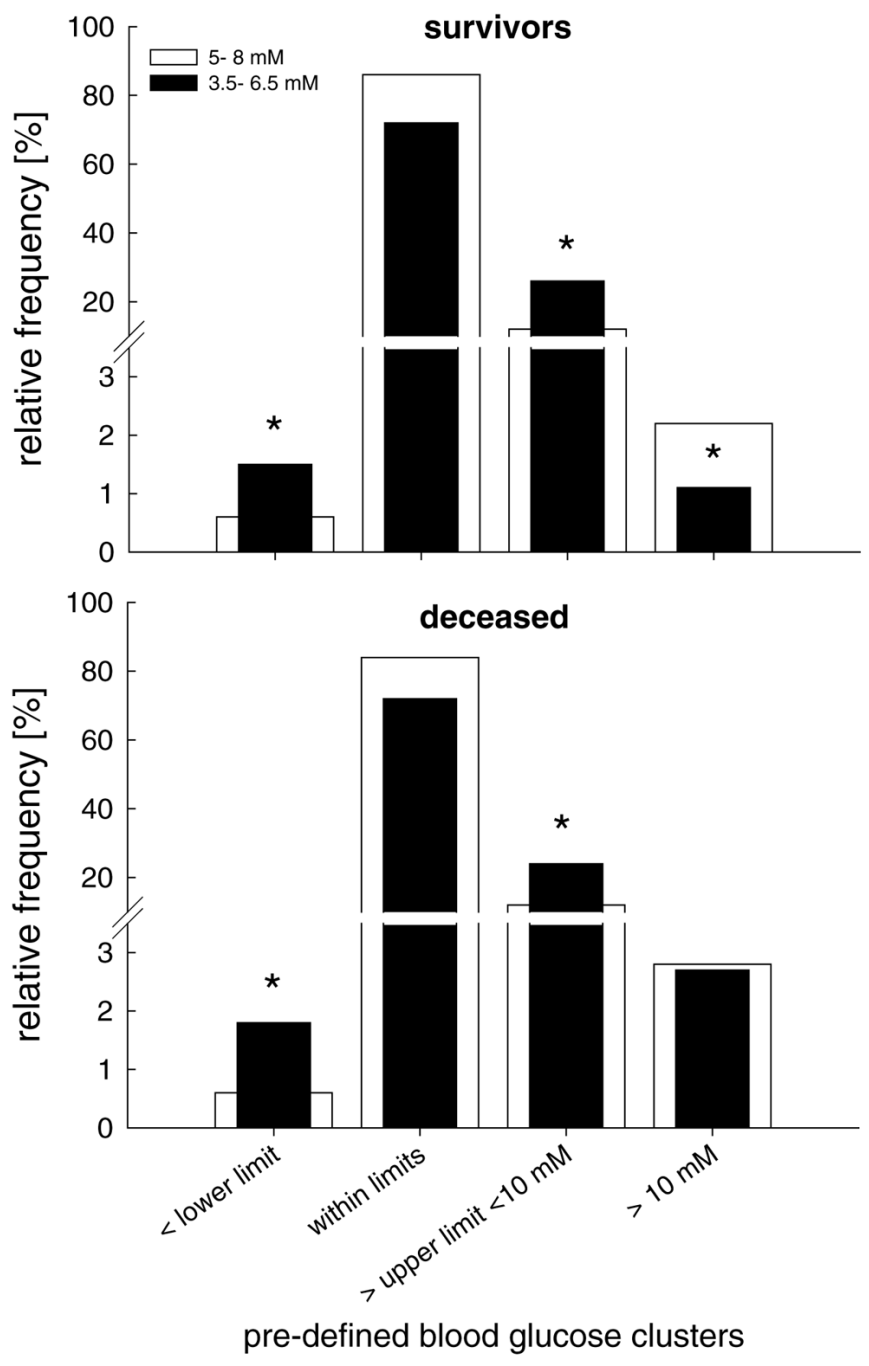
Frequencies of arterial blood glucose within target range. Shown are the relative frequencies of arterial blood glucose concentrations within the specified ranges, determined at 4-hour intervals. The frequencies of blood glucose levels below and above the predefined blood glucose target values were significantly increased in the 3.5 to 6.5 mmol/l compared with the 5 to 8 mmol/l group in the surviving patients (upper panel) and the patients who died (lower panel). In both groups, the majority of blood glucose values were within the target range. *P < 0.05, Mann-Whitney rank-sum test.

Overall, calculated relative frequencies in blood glucose values (number of values per pre- defined cluster expressed in percent of the total number) exhibited a normal distribution in surviving and deceased patients, regardless of treatment group, with maximal values at 5 to 5.9 mmol/l (5.9 ± 0.02 mmol/l) versus 6 to 6.9 mmol/l (6.8 ± 0.01 mmol/l) in the blood glucose targets 3.5 to 6.5 mmol/l and 5 to 8 mmol/l, respectively (Figure 3). The majority of blood glucose levels remained within the targeted blood glucose limits in surviving and deceased patients, irrespective of blood glucose target (Figure 3). Blood glucose levels below the lower limits (3.5 and 5 mmol/l, respectively) and above the upper limit (> 6.5 and > 8 mmol/l but remaining < 10 mmol/l) were predominantly found in the 3.5 to 6.5 mmol/l group (Figure 4, and Tables 2 and 3).

**Table 2:** Episodes of blood glucose levels below the lower limit

Survival status	Parameters	Blood glucose 3.5 to 6.5 mmol/l	Blood glucose 5 to 8 mmol/l
Survived	Blood glucose (mmol/l; median [range])	3.2 (0.7–3.4); NS	3.5 (1.5–3.9)
	Blood glucose < lower limit (*n *[%])	47/85 (55%)*	24/92 (26%)
	Episodes (median [range])	1 (1–6)	1 (1–11)
	Time point of occurrence (%)		
	Week 1	55%*	28%
	Week 2	24%	36%
	Week 3	21%	36%

Died	Blood glucose (mmol/l; median [range])	2.7 (0.6–3.4); NS	3.7 (3.1–3.9)
	Blood glucose < lower limit (*n *[%])	12/29 (41%)*	6/22 (27%)
	Episodes (median [range])	1.5 (1–6)	1.5 (1–3)
	Time point of occurrence (%)		
	Week 1	95%*	50%
	Week 2	5%	30%
	Week 3	0	20%

Shown are episodes of blood glucose levels below the lower limit in surviving patients and those who died within predefined blood glucose groups. Decreased blood glucose was predominantly encountered in the low blood glucose group during the first week. *P < 0.05, Whitney- Mann rank-sum test. NS, not significant.

**Table 3:** Episodes of blood glucose levels exceeding the upper limit

Survival status	Parameters	Blood glucose 3.5 to 6.5 mmol/l	Blood glucose 5 to 8 mmol/l
Survived	Blood glucose (mmol/l; median [range])	7.3 (6.6–14.8)*	8.7 (8.1–18.1)
	Blood glucose < lower limit (*n *[%])	81/85 (95%)	89/92 (97%)
	Episodes (median [range])	17 (2–75)*	8.5 (1–85)
	Time point of occurrence (%)		
	Week 1	55%	50%
	Week 2	24%	27%
	Week 3	21%	23%

Died	Blood glucose (mmol/l; median [range])	2.7 (0.6–3.4); NS	3.7 (3.1–3.9)
	Blood glucose < lower limit (*n *[%])	28/29 (97%)	20/22 (91%)
	Episodes (median [range])	11.5 (1–31)*	6.5 (1–61)
	Time point of occurrence (%)		
	Week 1	78%*	47%
	Week 2	16%	18%
	Week 3	6%	35%

Episodes of blood glucose levels exceeding the upper limit in surviving patient and those who died within the two predefined blood glucose groups. Increased incidences in elevated blood glucose levels were predominantly encountered in the low blood glucose group during the first week. *P < 0.05, Whitney-Mann rank-sum test.

The overlapping blood glucose levels result from maintaining arterial blood glucose levels within predefined tight limits of 3.5 to 6.5 mmol/l and 5 to 8 mmol/l. In both groups insulin was administered to reach the predefined glucose limits. The resulting overlapping range is 5 to 6.5 mmol/l. In surviving as well as deceased patients treated within the 3.5 to 6.5 mmol/l target, 52% of arterial blood glucose values were overlapping whereas 41% of arterial blood glucose values were overlapping in the 5 to 8 mmol/l target.

Severely hypoglycaemic values under 2.5 mmol/l were rare but mainly occurred in the 3.5 to 6.5 mmol/l rather than in the 5 to 8 mmol/l group (0.27% versus 0.027%; *P *> 0.001), corresponding to 14 versus three patients (12% versus 2.6%; *P *< 0.001). Hypoglycaemia mainly occurred during the first week (77%). Hyperglycaemic values exceeding 10 mmol/l were found in fewer than 3% of all measured blood glucose values, being significantly decreased in surviving patients within the 3.5 to 6.5 mmol/l group (Figure 4) and mainly encountered during the first week (75%).

### Blood glucose variability

In surviving patients blood glucose variability, determined by subtracting arterial blood glucose from previous values, was significantly greater in the 3.5 to 6.5 mmol/l group for blood glucose levels below the lower limit (3.5 mmol/l versus 5 mmol/l): -3.7 ± 0.2 versus -2.5 ± 0.4 (Mann-Whitney rank-sum test; *P *= 0.006). This was also the case for blood glucose levels within the limits (3.5 to 6.5 mmol/l versus 5 to 8 mmol/l): -0.43 ± 0.02 versus -0.22 ± 0.01 (Mann-Whitney rank-sum test; *P *< 0.001). For glucose levels exceeding the upper limit (6.5 mmol/l versus 8 mmol/l) there was no significant difference (1.4 ± 0.04 versus 1.4 ± 0.06; not significant).

In patients who died blood glucose variability was significantly different only for blood glucose levels within the predefined limits 3.5 to 6.5 mmol/l versus 5 to 8 mmol/l: -0.4 ± 0.05 versus -0.25 ± 0.03 (Mann-Whitney rank-sum test; *P *= 0.026). Below the lower and above the upper limit, there was no significant difference in blood glucose variability (below the lower limit [3.5 mmol/l versus 5 mmol/l]: -3.3 ± 0.6 versus -2.5 ± 0.6, not significant; above the upper limit [6.5 mmol/l versus 8 mmol/l]: 1.6 ± 0.1 versus 1.4 ± 0.1, not significant).

### Incidences and time points of decreased blood glucose levels

In surviving patients within the 3.5 to 6.5 mmol/l group there was a significant increase in two and three or more episodes of blood glucose levels below the lower limit as compared with the 5 to 8 mmol/l group (Table 2). These incidences predominantly occurred during the first week in the 3.5 to 6.5 mmol/l group (Table 2).

In deceased patients, reduced blood glucose levels below the lower limit were mainly encountered during the first week (Table 2).

### Incidences and time points of elevated blood glucose levels

In surviving patients and those who died within the 3.5 to 6.5 mmol/l group, there was a significant rightward shift toward increased frequency of sustained episodes of blood glucose levels exceeding the upper limit (Table 3), which was predominantly encountered during the first week.

### Changes in administered insulin and norepinephrine

**Figure 5:**
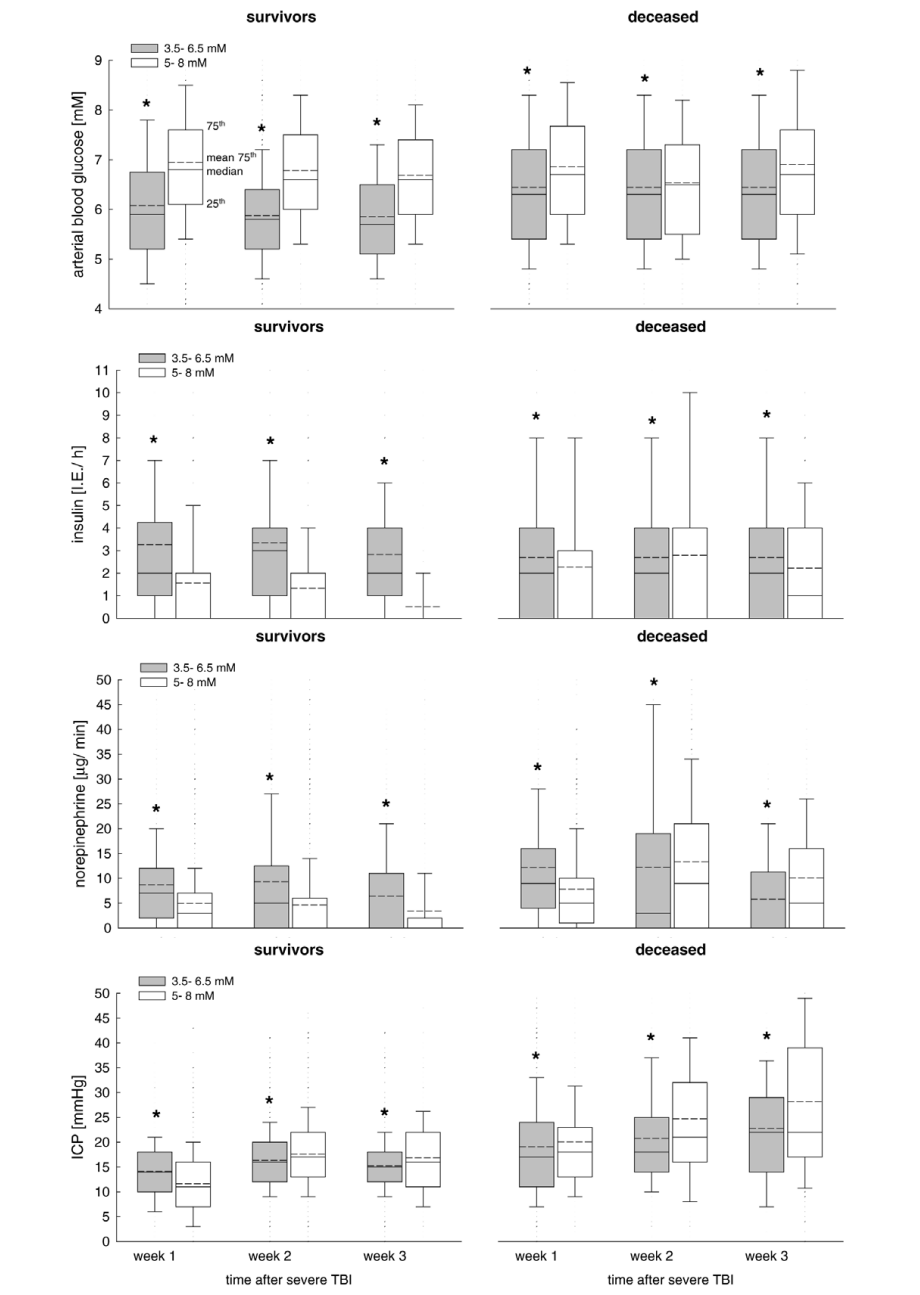
Changes in arterial blood glucose, insulin and norepinephrine dose, and ICP. Shown are changes in arterial blood glucose, insulin and norepinephrine dose, and intracranial pressure (ICP) in surviving patients and in those who died, within the different blood glucose target groups over time. **(a) **Arterial blood glucose levels were significanlty decreased in both surviving and deceased patients in the 3.5 to 6.5 group. **(b) **Insulin requirement was significantly increased in the 3.5 to 6.5 mmol/l group. **(c) **Within the 3.5 to 6.5 mmol/l group, surviving patients and those who died required significantly greater amounts of norepinephrine. **(d) **ICP was significantly increased in the 3.5 to 6.5 mmol/l group during the first week in surviving patients, followed by a significant decrease during the subsequent weeks. Patients who died exhibited a significantly increased ICP in the first week, irrespective of blood glucose target. In the third week, however, ICP was significantly increased in the 5 to 8 mmol/l group. *P < 0.05, analysis of variance on ranks.

Throughout the study period, surviving patients within the 3.5 to 6.5 mmol/l group (Figure 5a) required significantly more insulin (3.2 ± 0.04 versus 1.2 ± 0.03 units/hour; *P *< 0.001; Figure 5b) and norepinephrine (8.3 ± 0.1 versus 4.4 ± 0.08 μg/minute; *P *< 0.001; Figure 5c) compared with the 5 to 8 mmol/l group. This was less pronounced in the deceased patients.

### Changes in intracranial pressure and cerebral perfusion pressure

In surviving patients with targeted blood glucose levels between 3.5 and 6.5 mmol/l, ICP was significantly increased during the first week (14 ± 0.1 mmHg versus 12 ± 0.1 mmHg; *P *< 0.001) and significantly decreased during the third week compared with the 5 to 8 mmol/l group (15 ± 0.1 mmHg versus 17 ± 0.1 mmHg; *P *< 0.001; Figure 5d). Overall, deceased patients exhibited significantly increased ICP levels compared with surviving patients. In the deceased patients, elevated ICP levels were also significantly reduced in the 3.5 to 6.5 mmol/l group versus the 5 to 8 mmol/l group during the third week (22 ± 1 versus 28 ± 1 mmHg; *P *= 0.046; Figure 5d).

Overall, the incidence of elevated ICP of 20 mmHg or greater was comparable in the two blood glucose target groups and corresponding subgroups (survival versus death; 3.5 to 6.5 mmol/l versus 5 to 8 mmol/l: survivors 31% versus 40%; deceased 69% versus 60%)]. From the second week, however, the incidence of ICP of 20 mmHg or greater was significantly decreased in the patients who died within the low blood glucose target group (3.5 to 6.5 mmol/l versus 5 to 8 mmol/l: 24% versus 35% [week 2] and 23% versus 33% [week 3]). In surviving patients there was no difference.

Overall, CPP was maintained between 70 and 90 mmHg, without a clear influence of the different target blood glucose levels in surviving patients and those who died (data not shown).

### Impact of blood glucose diverging from the anticipated blood glucose targets

Higher blood glucose levels were associated with higher insulin requirement. Overall, blood glucose values above the upper limit or below the lower limit were not associated with an increase in ICP or a decrease in CPP (data not shown).

### Caloric intake

Average daily total caloric intake was comparable in both groups (3.5 to 6.5 mmol/l versus 5 to 8 mmol/l): 1,965 ± 38 versus 2,049 ± 35 kcal. There was no significant difference between the two groups on any given day.

### Bacteraemia, urinary tract infection, positive tracheobronchial secretions and blood inflammation parameters

Overall there was no statistically significant difference in rate of pneumonia between the two blood glucose groups. However, bacteraemia (25% versus 18%; relative difference: +28%), and urinary tract infections (22% versus 16%; relative difference: +27%) were significantly increased in patients within the 3.5 to 6.5 mmol/l group.

Over time, the rate of bacteraemia was not significantly different between the two blood glucose groups. The rate of pneumonia was significantly reduced in the third week in surviving and deceased patients within the 3.5 to 6.5 mmol/l group as compared with the 5 to 8 mmol/l group (18% versus 26%; -44%; *P *< 0.005). The rate of urinary tract infections was significantly decreased in the second week (26% versus 53%; -51%; *P *< 0.005) followed by a significant increase in the third week (48% versus 24%; +50%; *P *< 0.005) in patients within the 3.5 to 6.5 mmol/l group as compared with the 5 to 8 mmol/l group.

Within the 3.5 to 6.5 mmol/l group, bacteraemia was significantly less likely to be caused by Gram-positive bacteria (62% versus 78%; -26%; *P *< 0.05), whereas urinary tract infections were significantly more likely to be caused by Gram-positive bacteria (30% versus 17%; relative difference: +43%; *P *< 0.005) compared with the 5 to 8 mmol/l group. Gram-negative bacteria exhibited a similar rate in the two glucose groups. Tracheobronchial cultures revealed a similar distribution in Gram-positive and Gram-negative bacteria.

There were no differences in maximal leukocyte, CRP and IL-6 levels between the predefined blood glucose groups (data not shown).

## Discussion

In 228 propensity-matched patients suffering from severe TBI, the target blood glucose concentration of 3.5 to 6.5 mmol/l was associated with a trend toward increased mortality during the first 2 weeks, markedly increased frequency of hypoglycaemic and hyperglycaemic episodes, significantly elevated ICP during the first week, and markedly increased insulin and norepinephrine requirement compared with patients with a blood glucose target of 5 to 8 mmol/l. From the second week, however, decreased ICP and reduced rate of infectious complications prevailed in the 3.5 to 6.5 mmol/l group compared with the 5 to 8 mmol/l target group.

While a slightly higher blood glucose target (5 to 8 mmol/l) appears to be more beneficial during the first week, lower blood glucose levels (3.5 to 6.5 mmol/l) perhaps should be implemented during the first week.

### Limitations of this retrospective study

The present retrospective study is weakened by its lack of controlling for clinically important interventions, because investigated parameters were 'only' documented in 4-hour intervals or once daily. Thus, this approach is unfortunately likely to miss potentially important alterations that might have occurred within the 4-hour intervals. In addition, the present data do not allow us to assess the impact of speed and magnitude of blood glucose level correction, which might also be disadvantageous. To avoid this methodological setback, continuous recording and painstaking documentation of important events is required; this, however, is time consuming and difficult in the daily routine.

Our assimilation of patients recruited during sequential time periods (2000 to 2002 versus 2002 to 2004) by pre-defining age, sex, as well as presence and severity of additional injuries allowed us to control for certain baseline variables, thereby enhancing the quality of our retrospective analysis of pooled data within *post hoc *defined clusters. Normalization of the data by calculating relative frequencies within predefined clusters helps to compare patient groups and permits determination of the potential impact of blood glucose targets. However, we cannot exclude the possibility that improved awareness and knowledge, which clearly develop over time, might also have influenced basic treatment and could have blurred relevant differences.

Owing to differences in individual clinical course and different durations of hospitalization, patients exhibit different values for the various parameters; this may account for the reduced number of values recorded the third week, especially in the patients who died. Thus, we obtained the greatest statistical power within the first and second weeks.

The chosen blood glucose targets are overlapping. Thus, the close proximity of the upper and lower limits of the two blood glucose targets, namely 6.5 and 5 mmol/l, might have obscured an even more significant impact, as in the study published by van den Berghe and colleagues [7], when larger differences were studied under 6.1 mmol/l versus under 12 mmol/l. However, in reality, even in that prospective study, the difference between low and high blood glucose target groups (< 6.1 versus < 12 mmol/l) was much smaller, being on average 5.6 mmol/l versus 8.9 mmol/l, with similar initial blood glucose values [7,8]. The rate of overlapping blood glucose values, however, was not reported [7,8,15].

The overlapping values resulting from insulin administration, and which are a reflection of the meticulous attention given to adhering to the predefined blood glucose targets in both groups, appear to have reduced the impact in the present study. However, the significant differences in primary end-points, glucose variability and extreme blood glucose values show that the predefined blood glucose targets are of pathophysiologic relevance, despite overlapping of blood glucose values. Within this context, patients within the 3.5 to 6.5 mmol/l group were metabolically less stable, as reflected by the higher incidence of hypoclycaemic and hyperglycaemic vlaues. Apparently, the chosen lower limit of 3.5 mmol/l predisposes to hypoglycaemic complications in the face of suppressed hormonal counterregulation. However, as was recently demonstrated by McMullin and colleagues [14], who compared the target range 5 to 7 mmol/l versus 8 to 10 mmol/l, similar difficulties were encountered even at higher blood glucose targets.

### Blood glucose and secondary brain damage

TBI is characterized by regionally and temporally altered glucose metabolism caused by altered cellular demands and functional disturbances. These changes are not restricted to the site of injury [16,17] and can persist for up to several months in patients with moderate to severe TBI [18-21].

In face of the limited cerebral energetic reserves, with marginal cerebral availibility of glycogen, glucose is the predominant fuel for neuronal and glial activities [22]. To ensure adequate glucose supply in the face of increased glucose consumption, cerebral glucose uptake occurs independently of insulin via specific endothelial/glial (glucose transporter [GLUT]1) and neuronal (GLUT3) glucose transporters, which have different transport characteristics. In this context, GLUT1 (with its intermediate Michaelis constant of 5 to 7 mmol/l) and GLUT3 (with its low Michaelis constant of 1.6 mmol/l) ensure neuronal glucose uptake even during hypoglycaemia [23]. Nevertheless, any decrease in blood glucose levels, such as those observed in the present study, predisposes the patient to risk for reaching the lower glucose transportation rate, especially in endothelial/glial glucose transporters, which can be aggravated by concomitant impaired perfusion and sustained glycolysis [24] or altered enzymatic activity [20,21]. This, in turn, increases the risk for additional injury. In this regard, a decrease in blood glucose levels below 8 mmol/l was associated with an increase in extracellular cerebral lactate, measured using microdialysis, which coincided with a significant elevation in perischaemic cortical depolarizations [12]. A dramatic increase in perischaemic cortical depolarizations was observed when blood glucose levels dropped below 6 mmol/l [11-13]. By implementing low blood glucose levels (such as 3.5 to 6.5 mmol/l [present study] or 4.4 to 6.1 mmol/l [7]), we are actively risking progressive and additional secondary insults, which could aggravate underlying structural and functional damage. Evidence for such a process was provided by Vespa and colleagues [25], who reported a significant increase in glutamate and lactate/pyruvate ratio during intensive insulin therapy with arterial blood glucose levels ranging from 5 to 6.7 mmol/l versus 6.7 to 8.3 mmol/l.

In addition, hypoglycaemia combined with insufficient tissue oxygenation predisposes the brain to aggravated damage induced by subsequent hyperglycaemia [26]. The significant increase in ICP and elevated requirement for norepinephrine to maintain CPP above 70 mmHg observed in the present analysis could reflect ongoing alterations within the injured brain, possibly induced by maintaining blood glucose levels between 3.5 and 6.5 mmol/l, because this range is close to the threshold for inducing cortical spreading depressions with subsequent oedema progression [13]. The significant increase in ICP coincided with an increase in hypoglycaemic values, which were predominantly observed during the first week. Because the majority of pathological cascades are activated within the first week, any additional insults, such as hypogycaemia, hyperglycemia and changing blood glucose values, should be avoided to prevent secondary brain damage.

Apart from hypoglycaemia-induced damage, hyperglycaemia is also a feared complication for its detrimental effects. In this context, hyperglycaemia has the following effects [27-29]: it impairs cerebral perfusion because of cellular swelling or neutralization of nitric oxide by free radical production; it promotes local tissue acidosis; it induces oxidative stress with subsequent mitochondrial damage and impaired oxidative phosphorylation; it promotes glutamate-driven increase in intracellular calcium concentrations; it induces microcirculatory damage and blood-brain barrier disruption because of elevated inflammation with sustained cerebral leukocyte adherence and invasion, and production of matrix metalloproteinase-9; and it interferes with transcription processes.

The general consensus is to avoid blood glucose levels exceeding 10 mmol/l, because they are associated with neurologic deterioration [23]. In the present study, dangerously elevated blood glucose levels exceeding 10 mmol/l were observed in fewer than 3% of all blood glucose values. Neither these hyperglycaemic nor the hypoglycaemic values were associated with signs of cerebral worsening (increased ICP or decreased CPP).

### Pharmacodynamic effects of insulin

Insulin is known for its anabolic effects, which promote lipogenesis and protein synthesis mediated by uptake of glucose and amino acids. In addition, insulin inhibits hyperglycaemia-induced oxidative cell damage [6,7,27], thereby positively influencing various intracellular signaling cascades [30]. These effects are viewed as the pharmacodynamic basis of improved organ function: decreased renal failure [31], reduced ventilatory support [32], decreased infection rate [33] and reduced transfusion requirements [31]. They contribute to shortened length of ICU stay [32], improved recovery and decreased mortality [32] in critically ill patients [7,8]. These positive findings are in contrast to the present findings of sustained haemodynamic instability and increased infections (bacteraemia and urinary tract infections) in patients subjected to the low blood glucose target (3.5 to 6.5 mmol/l versus 5 to 8 mmol/l). This, of course, could result from differences in policy concerning volume management (type, duration, or predefined targets), catheter placement (duration before renewal or removal) and administration of antibiotics (type, duration and start), because we maintained similar blood glucose levels using similar insulin doses as were reported by van den Berghe and coworkers [7,8]. At the a cellular level, insulin attenuates norepinephrine-mediated vasoconstriction [34], which could explain the increased norepinephrine requirement in patients within the 3.5 to 6.5 mmol/l group. In addition, attenuated respiratory burst [35] and decreased chemokinesis [36] in neutrophils caused by insulin-mediated decreased blood glucose levels contribute to impaired cellular defense mechanisms, thereby promoting infections, as was observed in the present study in terms of increased bacteraemia and urinary tract infections. A concentration-dependent effect in critically ill patients requires investigation.

### Control of blood glucose levels

Implementing a strict and tight control of blood glucose levels by intensified insulin administration has been shown to result in more rapid correction, more stable blood glucose concentrations and longer maintenance within the predefined target range as compared with controls [37]. This, however, requires steady parenteral or enteral supply along with nutrients, and tight control to prevent hypoglycaemic episodes, which are feared for their cerebrotoxic effects.

van den Berghe and colleagues [7] used a particular protocol, which includes continuous infusion of glucose (9 g/hour), intake of sufficient calories (19 kcal/kg per hour) during continuous insulin administration (0.04 units/kg per hour). In our patients subjected to a blood glucose target of 3.5 to 6.5 mmol/l, a similar insulin dose was administered to that reported by van den Berghe and coworkers [7,8]. Apparently, glucose must be co-infused to prevent and correct insulin-induced hypoglycaemia. This, however, was not done in our patients with severe TBI, because infusing glucose results in administration of 'free' water, which could aggravate brain oedema formation because glucose and free water can diffuse more easily and rapidly through the blood-brain barrier compared with crystalloids.

Regardless of strategy, maintaining glucose within predefined limits can be difficult, with hypoglycaemic episodes ranging from 12% to 40% in patients subjected to intensified insulin treatment compared with 1.2% to 7.4% in the conventional group [7-9,14,33,38-40]. This is in line with the observed global incidence of hypoglycaemic values (< 2.5 mmol/l), occurring in 12% (14/114 patients; 3.5 to 6.5 mmol/l group) versus 2.6% (3/114 patients; 5 to 8 mmol/l group) in the presently investigated 228 patients suffering from severe TBI. These data also clearly show that intermittent control in 4-hour intervals is insufficient to prevent increased or decreased blood glucose levels. Continuous control would yield superior results. However, an adequate procedure has not yet been introduced into clinical routine practice.

### Are there different requirements for specific time points following severe TBI?

In daily clinical routine, patients undergo different phases that require different types and degrees of interventions. Although haemodynamic instability and systemic inflammation prevail during the first week, infections with a new inflammatory challenge develop during the second week. Some patients again recover without additional complications. Thus, an individual strategy might be required to maintain metabolic stability and prevent additional insults related to blood glucose deviations that might result in increased mortality. These dynamic and time-dependent processes could also influence the threshold for additional cell damage over time.

Based on the present data (ICP, length of ICU stay, infections, mortality, and hypoglycaemic and hyperglycemic episodes), patients do not profit from blood glucose levels maintained between 3.5 and 6.5 mmol/l during the first week. From the second week, however, lowered blood glucose levels could prove beneficial because ICP levels were significantly decreased compared with patients in whom blood glucose levels were maintained between 5 and 8 mmol/l. However, ICP values were in a similar range, making it difficult to assess positive effects at the bedside in real time. Such a possible time-dependent pattern is also suggested by the findings reported by van den Berghe and coworkers [7], wo reported that patients only profit from lowered blood glucose levels achieved using an intensified insulin therapy if they require ICU treatment longer than 3 days (medical ICU patients) or 5 days (surgical ICU patients) [15]. In fact, mortality was significantly increased in medical patients remaining on the ICU for fewer than 3 days [15]. In critically ill patients suffering from isolated TBI and subjected to low blood glucose levels (average 5.6 ± 0.5 mmol/l), mortality on the ICU at 6 and 12 months was increased (23% versus 18%, 48% versus 30%, respectively) [8].

This could be in favour of a certain beneficial effect of elevated blood glucose levels during the early phase following a defined insult, as suggested by Ghandi and colleagues [41] and as is also seen under *in vitro *conditions where short-term hyperglycaemia (15 to 60 mmol/l) is protective in cardiac myocytes, astrocytes and neurons [42-44].

Future studies are required to investigate dynamic adaptation of blood glucose targets over time.

## Conclusion

Based on our retrospective analysis, revealing a significant increase in hypoclycaemic and hyperglycemic episodes, as well as elevated insulin and norepinephrine requirements, we cannot recommend maintaining blood glucose levels between 3.5 and 6.5 mmol/l during the first week after severe TBI. Maintaining arterial blood glucose between 5 and 8 mmol/l is more favourable during the first week. However, significant decreases in ICP, including intracranial hypertension exceeding 20 mmHg, as well as reduced infectious complications during the second week are in favour of the lower arterial blood glucose levels maintained between 3.5 and 6.5 mmol/l.

It remains to be determined whether a temporally adapted blood glucose target might be required. For this, however, an optimal blood glucose level must be defined. In addition, surveillance of changes in blood glucose needs to be improved, preferably using continuous measurements, because intermittent analysis is limited by its risk for delayed assessment and correction of hypoglycaemic and hyperglycemic episodes. Furthermore, downstream monitoring of metabolic impairment, as indicated by parameters such as cerebral lactate, lactate/pyruvate ratio, glucose/lactate ratio and glutamate level, is indispensible in identifying adequate and optimal blood glucose level, which is required for subsequent decision making and treatment.

## Key messages

• Maintaining low blood glucose concentrations between 3.5 and 6.5 mmol/l, as compared with 5 to 8 mmol/l, increases rate of hypoglycaemic and hyperglycaemic values, especially during the first week.

• The need for norepinephrine to maintain stable CPP level is significantly increased when the target blood glucose level is low.

• The rates of bacteraemia and urinary tract infection are significantly increased when reducing blood glucose levels to 3.5 to 6.5 mmol/l as compared with 5 to 8 mmol/l during the first week, followed by a significant decrease in the second week.

• Temporal profile of decreased ICP suggests that blood glucose levels maintained between 3.5 and 6.5 mmol/l could be of benefit during the second week, reflected by decreased incidence of ICP exceeding 20 mmHg.

• Future trials must determine a characteristic temporal profile of specific arterial blood glucose targets, because the present study suggests less favourable effects of low blood glucose levels (3.5 to 6.5 mmol/l) during the first week, followed by more favourable effects as of the second week compared with the blood glucose target of 5 to 8 mmol/l.

## Abbreviations

CPP = cerebral perfusion pressure; CRP = C-reactive protein; GLUT = glucose transporter; ICP = intracranial pressure; ICU = intensive care unit; TBI = traumatic brain injury.

## Competing interests

The authors declare that they have no competing interests.

## Authors' contributions

RM collected most of the data, drafted parts of the manuscript and performed graphical analysis. MB helped analyzing and interpreting the data, and drafted parts of the manuscript. SL and JS were responsible for data collection and upkeeping of the databank. MK, PS and RS helped to analyze and interpret the data. JFS conceived the study design, collected some of the data, performed graphical and statistical analyses, and drafted parts of the manuscript.
